# Decoding GuaB: Machine Learning-Powered Discovery of Enzyme Inhibitors Against the Superbug *Acinetobacter baumannii*

**DOI:** 10.3390/ph18121842

**Published:** 2025-12-02

**Authors:** Mohammad Abdullah Aljasir, Sajjad Ahmad

**Affiliations:** 1 Department of Medical Laboratories, College of Applied Medical Sciences, Qassim University, Buraydah 52571, Saudi Arabia; mjasr@qu.edu.sa; 2 Department of Health and Biological Sciences, Abasyn University, Peshawar 25000, Pakistan

**Keywords:** machine learning, molecular docking, antimicrobial resistance (AMR), MMGB/PBSA, RDF

## Abstract

**Background/Objectives:** GuaB, which is known as inosine 5′-phosphate dehydrogenase (IMPDH), is an enzymatic target involved in the de novo guanine biosynthetic pathway of the multidrug-resistant (MDR) *Acinetobacter baumannii*. GuaB has emerged as a potential therapeutic target to cope with increasing antibiotic resistance. Here, we used machine learning-based virtual screening as a verification technique to find potential inhibitors possessing different chemical scaffolds, using structure-based drug design as a discovery platform. **Methods:** Four machine learning models, built based on chemical fingerprint data, were trained, and the best models were used for virtual screening of the ChEMBL library, which covers 153 active molecules. Molecular dynamics (MD) simulations of 200 ns were carried out for all three compounds in order to explain conformational changes, evaluate stability, and provide validation of the docking results. Post-simulation analyses include principal component analysis (PCA), bond analysis, free-energy landscape (FEL), dynamic cross-correlation matrix (DCCM), radial distribution function (RDF), salt-bridge identification, and secondary-structure profiling, etc. **Results:** For molecular docking, the screened compounds were used against the GuaB protein to achieve proper docked conformation. Upon visual examination of the best-docked compounds, three leads (lead-1, lead-2, and lead-3) were found to have better interaction with the GuaB protein in comparison to the control. The mean RMSD scores between the three leads and the control were between 2.54 and 2.89 Å. In addition, the three leads as well as the control were characterized for pharmacokinetic features. All three leads met Lipinski’s Rule 5 and were thus drug-like. PCA and FEL analyses showed that lead-2 exhibited improved conformational stability, identified as deeper energy minima, whereas RDF and DCCM analyses revealed that lead-2 and lead-3 exhibited strong local structuring and concerted dynamics. In addition, lead-2 displayed a very rich hydrogen-bonding network with a total of 460 frames possessing such interactions, which is the highest among the complexes investigated here. Based on entropy calculations and the maximum entropy method of gamma–gram, lead-1 proved to be the most stable one with the lowest binding free-energy. **Conclusions:** This study provides an integrated machine learning-based virtual screening pipeline for the identification of new scaffolds to moderate infections associated with AMR; however, in vitro validation is still required to assess the efficacy of such compounds.

## 1. Introduction

Antimicrobial resistance, commonly referred to as antibiotic resistance or AMR, has become a significant and catastrophic problem that has increased healthcare systems’ expenses globally [1]. An estimated 1.3 million people die from AMR each year, making it one of the most serious threats to human health [2]. Significant morbidity, death, and higher costs have been linked to it in recent years due to the longer hospital stays and treatment duration. Despite the addition of new antimicrobial medicines to the global toolbox in recent decades, resistance appears to be a growing issue with geometric progression [3]. An estimated 8500 cases and 700 fatalities were caused by carbapenem-resistant *Acinetobacter* spp.-related diseases in the United States in 2017. These figures surged in 2020 (7500 cases, 700 deaths), while they declined in 2018 (6300 reported cases, 500 deaths) and remained constant in 2019 (6000 cases, 500 deaths). According to preliminary research by the Centers for Disease Control and Prevention (CDC), hospital-onset carbapenem-resistant Acinetobacter-species infections increased by 78% between 2019 and 2020 [4].

The ESKAPE organisms (*Enterococcus faecium*, *Staphylococcus aureus*, *Klebsiella pneumoniae*, *A. baumannii*, *Pseudomonas aeruginosa*, and *Enterobacter* spp.) include *A. baumannii* [5]. The organism *A. baumannii* is oxidase-negative, aerobic, Gram-negative, non-fermentative, and non-motile. Despite the large number of species that have been identified, *A. baumannii* is the most medically relevant. The organism is endemic in both the water and the soil environments. It is implicated in an assortment of patient infections that emerge from soft tissues, from open wounds, saliva, and urine [6]. The rise in prevalence of invasive procedures and the growing number of antibiotic-resistant strains, and a permanently increasing population of immunocompromised patients, have led to the spread of *A. baumannii* in the hospital milieu. This widespread distribution is due to the extreme survival status of the organism in terrible situations and an absurdly rich genetic repertoire, which allows for the acquisition of a wide range of resistance factors quickly. Multidrug-resistant Acinetobacter infections and outbreaks are, as such, a common occurrence and have been reported in the literature [7]. When exogenous guanine is not available for them, Gram-negative bacteria use the enzyme GuaB, called inosine 5′,-monophosphate dehydrogenase (IMPDH), as a simple life source. IMPDH catalyzes the first and rate-limiting step of de novo guanine nucleotide synthesis via the oxidation of inosine 5 5′-monophosphate (IMP) to xanthosine 5 5′-monophosphate (XMP). XMP is, in turn, recycled to guanosine 5′-monophosphate (GMP) by GMP synthase [8]. Consequently, blocking IMPDH leads to low intracellular levels of GTP and dGTP, the needed building blocks of RNA and DNA, respectively [9]. Thus, GuaB is a promising and emerging therapeutic target for the management of antimicrobial resistance-led infections.

One of the studies reported by [10] focused on GuaB inhibition with effectiveness against the *A. baumannii* diseases. They reported the identification of highly effective small-molecule GuaB inhibitors that are specific to pathogenic bacteria and have bactericidal activity in vivo against *A. baumannii* animal models of infection, with a small percentage of on-target spontaneous resistance. Multidrug-resistant bacteria, which are a top priority for the development of novel antibiotics, are part of the broad GuaB action. Co-crystal structures of GuaB proteins bound to inhibitors from *A. baumannii*, *E. coli,* and *S. aureus* reveal similar GuaB binding modes across species and pinpoint important binding site residues that predict whole-cell activity in both Gram-positive and Gram-negative bacterial clades. The small-molecule GuaB inhibitors’ obvious in vivo effectiveness in a bacterial infection model confirms GuaB’s status as a crucial antibiotic target.

Drug discovery is accelerated by machine learning (ML), which minimizes the time, expense, and significant failure rate of conventional methods by analyzing vast biological datasets to identify patterns, predict molecular features, screen candidates, and optimize drug designs [11]. By improving in silico (computer-based) research, enhancing lead candidate selection, identifying drug–drug interactions, and optimizing pharmacokinetic and pharmacodynamic (PK/PD) features, ML accelerates the conventional, drawn-out, and expensive drug discovery process [12]. With the ultimate objective of quicker, less expensive, and more effective treatments, uses include drug repurposing, protein structure evaluation for improved antibody design, and even the creation of novel therapeutic candidates from scratch [13]. There is potential for guided drug design based on ML in the drug discovery domain [14]. This method effectively directs the discovery and optimization of new drug candidates by utilizing machine learning algorithms. Lead compound identification and properties optimization become faster, and the entire drug discovery process is streamlined when machine learning is integrated into drug design pipelines. Though GuaB has emerged as a potential AMR therapeutic target, no systematic computational study integrating ML, docking, and MD simulation has been reported. This gap hinders the progress of targeted drugs against the bacteria. In the current study, ML and molecular dynamics methods were used in the prioritization of potential GuaB inhibitors as one approach to fighting AMR caused by *A. baumannii.* Lead compounds 1–3 are computationally predicted structures which are not synthesized, nor have they yet been tested experimentally against the bacteria. The inhibition of Guab stops guanosine biosynthesis, thereby aiding in the management of infections caused by *A. baumannii.* The ML-MD strategy adopted in the present research is comprehensive, as no such methodology has been used before for the inhibition of GuaB.

## 2. Results

### 2.1. Datasets Preprocessing

An integrated approach of cheminformatics and bioinformatics was implemented to find prospective GuaB inhibitors to treat *A. baumannii*-associated infections. Compounds were collected and were then fingerprinted and used for descriptor calculation. Duplicate entries and missing values were detected by using the quality assessment function, and the dataset was found free from duplicate entries and missing values and appropriate for analysis at the following stage. SMILES notation was converted into numerical descriptors during the preprocessing stage, which allowed us to statistically characterize each molecule. The dataset was then split for training and testing purposes, and, importantly, both processes contained an equal representation of active and inactive compounds.

### 2.2. Model Development, Implementation, and Validation

Active inhibitors of GuaB were categorized using the various machine learning methods, i.e., k-nearest neighbor (kNN), support vector machine (SVM), random forest (RF), and extreme gradient boosting (XGBoost). The selected dataset was used to train these models, which were created using the Python sklearn module v 1.7. Several statistical measures, such as specificity, accuracy, recall (sensitivity), AUC, and MCC, were used to assess these models’ efficacies. Table 1 shows how well each model performed on the test set.

**Figure 1:**
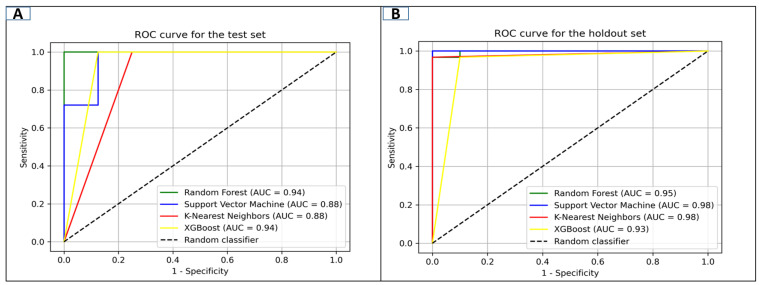
Representation of the receiver operating characteristic (ROC) curve for each model using the (**A**) test and (**B**) hold-out sets.

The performances of the ML models (test sets and hold-out sets) were assessed by different factors in Figure 1. The receiver operating characteristic (ROC) curve gives information about the abilities of the model regarding the separation of active and inactive compounds, whereas the area under the curve (AUC) assesses the estimated precision. The findings from the test datasets indicate that the RF and XGBoost models performed better than the others on all of the metrics. According to Figure 1, they obtained an AUC score of 0.94. However, the findings of the hold-out tests indicate that all models performed well, with the SVM and KNN models both achieving the top-ranked performance score of 0.98.

### 2.3. In Silico Molecular Docking Analysis

As a useful tool, a comprehensive docking analysis was performed to further clarify the process of interaction between compounds and the GuaB catalytic site. Structure-based virtual screening was carried out, sampling the GuaB protein of *Acinetobacter baumannii* densely. The analysis also grasped variations and conformational shifts in the complexes in docked complexes, including for protein–ligand interactions (Samykannu et al., 2024). The docking grid was focused on the Ca atoms in these residues, and the size of the grid was 30 × 30 × 30 Å, covering the targeted site completely. Flexibility of the ligands was enabled, and the exhaustiveness parameter was set to 12 to allow for an extensive search of the conformations. The ten compounds with the strongest binding affinities were selected for further investigation after a thorough screening of 153 compounds [15]. The molecular docking performances are displayed in a heatmap in Figure S1, with the binding affinity distribution profile (histogram and scattered plot). Most of the compounds were grouped between −6.5 and −5.3, indicating favorable binding. The complex molecule 3-(5-(3, 4-dimethylphenyl)-1, 2, 4-triazolidin-3-yl)-4-hydroxybenzenesulfonamide had the highest binding affinity of any compound. As shown in Table 2, the compound’s binding affinity was −8.1 kcal/mol. The second hit compound, 3-carboxy-1-(2,4-difluorophenyl)-6-fluoro-5-methyl-7-(piperazin-1-yl)quinolin-1-ium-4-olate, showed the binding affinity of −7.6 kcal/mol. Subsequently, the third hit compound, named 2-(5-(3,4-dimethylphenyl)-1,2,4-triazolidin-3-yl)-4,6-dimethylphenol, had the binding affinity of −7.5 kcal/mol. A known inhibitor of GuaB, 3-(hydroxymethyl)-6-((1-((4-methyl-3-morpholinophenyl)amino)-1-oxopropan-2-yl)amino)quinolin-1-ium, was used as the control in order to validate the docking protocol and benchmark the degree of ligand efficiency. This control has been chosen since its inhibitory activity and binding mode have been reported experimentally before, which enabled us to evaluate the reliability of the docking poses and evaluate the predicted binding energies of novel lead compounds in comparison to a validated inhibitor [16]. The control molecule (−7.5 kcal/mol) was used as a baseline assessment to measure the effectiveness of novel identified drugs. The docking score noted for the control was −7.5 kcal/mol, as shown in Table 2, along with the top-ranked compounds exhibiting promising binding affinities and stability at the active site of GuaB with non-covalent interactions.

**Figure 2:**
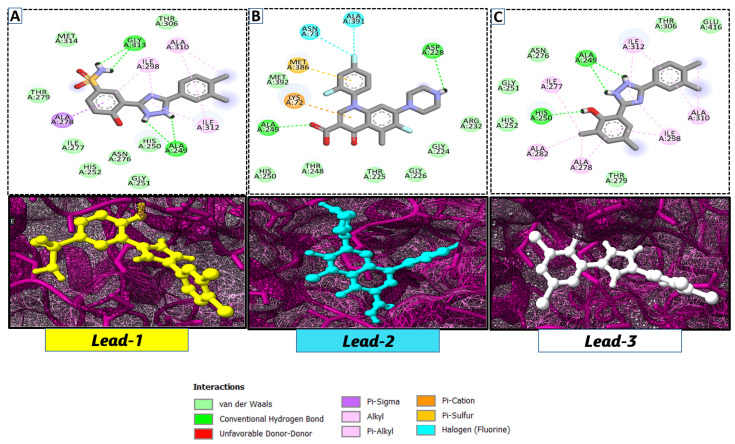
A binding view of all three novel compounds with GuaB. The top row exhibits the 2D interactions of identified compounds with the target protein, while the bottom row illustrates a 3D view of three leads, designated as lead-1 (**A**), lead-2 (**B**), and lead-3 (**C**).

**Figure 3:**
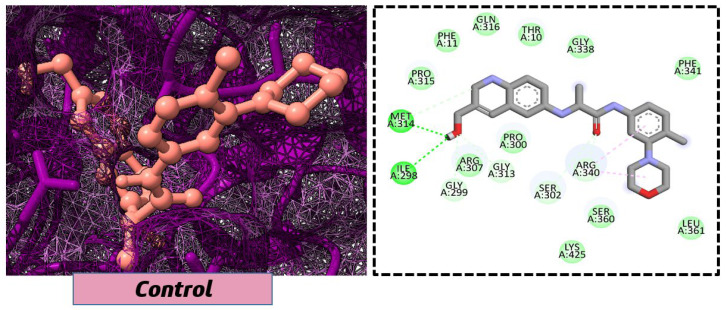
The binding mode of the control molecule with the GuaB target protein, both 2D and 3D illustrations.

Lead-1 (Figure 2A) showed specific h-bonds with Gly313 and Ala249 and van der Waals interactions with Met314, Thr279, Ile277, His252, Asn276, His250, Gly251, and Thr306. Moreover, π-alkyl interactions were seen with Ile298, Ala310, and Ile312. The second-ranked compound (Figure 2B) had hydrogen-bonding interactions with Asp228, Ala249, and Unk802, whereas van der Waals bonds were formed with the residues Met392, His250, Thr248, Thr255, Gly226, Gly224, and Arg232. In addition, the ligand ranked third (Figure 2C) formed hydrogen bonds with Ala249 and His250. On the other hand, the same ligand (Figure 2C) showed van der Waals interactions with the residues Glu416, Thr306, Gly251, Asn276, His252, and Thr279. Π-alkyl and alkyl bonds were formed with the residues Ile312, Ala310, Ile298, Ala278, Ala282, and Ile277, respectively, as depicted in Figure 2. The control molecule, which acted as a baseline compound/drug, exhibited hydrogen bonding with the GuaB residues Met314 and Ile298, as shown in Figure 3.

### 2.4. ADMET Properties and Lipinski Rule 5 Fulfillment Standards

The SwissADME online server was employed to evaluate the ADME properties of all shortlisted ligands, as detailed in Table 3. The molecular weights (MWs) of the lead compounds targeting GuaB varied between 297.39 g/mol and 417.38 g/mol Da, remaining below the generally recognized threshold of 525 Da. The MlogP score, which shows lipophilicity, was determined to be within the drug-like range of 1.21 to 3.08, as given in Table 3. All compounds demonstrated high GI and complied with Lipinski’s rule of five, indicating decent oral bioavailability potential. The compounds’ topological polar surface area (TPSA) values, which affect drug transport features, were between 56.32 Å^2^ and 124.86 Å^2^. The topological polar surface area (TPSA) values suggest promising membrane permeability, although indicating low permeation across the blood–brain barrier that may reduce possible side effects in the central nervous system (CNS) [17]. Moreover, the top three shortlisted compounds had no predicted AMES toxicity or hERG I and II inhibition, suggesting a good preliminary safety profile. The control compound, although non-mutagenic, showed potential hERG II inhibition, which triggers the potential of cardiotoxicity. Overall, the results suggest that the lead compounds that were chosen may have less risk of toxicity than the control.

### 2.5. Molecular Dynamics (MD) Simulation

MD simulation predicts potential atomic positions and analyzes their time-dependent behavior [15]. It is used to investigate the structure, movements, and thermodynamics of biological molecules and their complexes. To fully understand the structural stability and dynamic behavior of protein–ligand complexes, a variety of significant molecular dynamics parameters were examined, such as root-mean-square deviation (RMSD), root-mean-square fluctuation (RMSF), radius of gyration (Rg), principal component analysis (PCA), free-energy landscape (FEL), hydrogen-bond interactions, dynamic cross-correlation matrix (DCCM), secondary-structure transitions, and salt-bridge formation. RMSD, RMSF, and Rg were used to give an overview of stability, flexibility, and compactness at the global and residue level. PCA and FEL were further used to allow for the identification of predominant motions and energetic landscapes, and thus allow for the identification of stable conformational states caused by ligand binding. Analyses of hydrogen bindings and salt bridges emphasized the persistence and amplitude, and importance of key non-covalent interactions that are responsible for the stabilization of complexes. The DCCM and secondary-structure evaluation provided information on the correlated motions of residues and structural integrity over the course of the simulation. These analyses provide insights into the overall dynamic behavior of GuaB–ligand complexes.

#### 2.5.1. RMSD Alongside RMSF Studies

**Figure 4:**
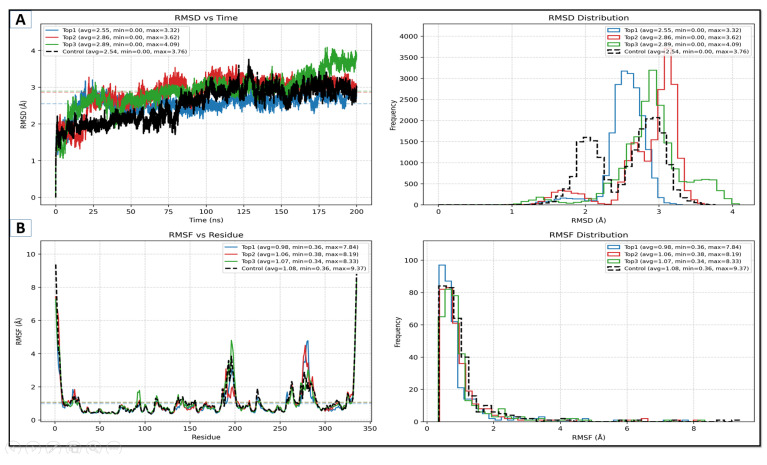
MD simulation trajectories analysis: RMSD (**A**) and RMSF (**B**) for novel lead complexes, designated as top1 (lead-1), top2 (lead-2), and top3 (lead-3), along with the control molecule.

Root-mean-square deviation (RMSD) and (RMSF), root-mean-square fluctuation, were used to measure protein–ligand stability and ligand residue fluctuations across time (Samad et al., 2023). Their distribution is indicative of the frequency of observed values and gives an idea of dynamic behavior. The mean values, along with the plot of lead complexes, can be seen in Figure 4, which demonstrates that top1, designated as lead-1, and the control remained structurally stable as compared to top2 and top3, which showed moderately high RMSD scores. On the other hand, the RMSF analysis depicted that the lead complexes show similar profiles, with the control exhibiting a higher maximum RMSF (9.37 Å). The lead complexes’ maximum scores noted were 7.84 Å, 8.19 Å, and 8.33 Å for Lead-1, Lead-2, and Lead-3.

#### 2.5.2. Rog and Beta Factor

**Figure 5:**
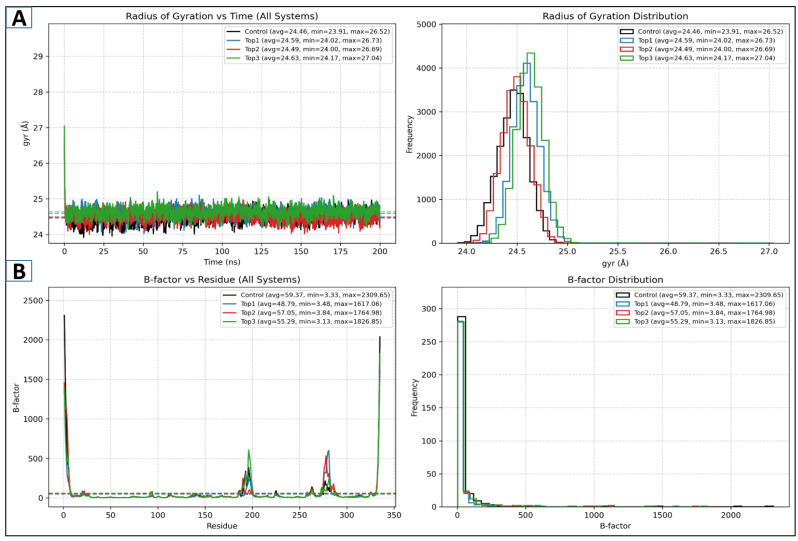
Assessment of the MD simulation trajectory plots: Rog (**A**) and beta factor (**B**) for lead complexes, designated as top1 (lead-1), top2 (lead-2), and top3 (lead-3), along with the control molecule.

In order to evaluate the docked complexes’ molecular stability and compactness, an additional analysis of the protein’s radius of gyration was carried out [18]. Figure 5A illustrates that the Rog values of the lead docked complexes between 24.46 and 24–63 Å demonstrate a higher level of compactness, remaining very stable over the course of a 200 ns MD simulation. These results demonstrate promising structural compactness over the simulation period [19]. Moreover, a beta-factor analysis was carried out to evaluate residue-level fluctuations in protein–ligand complexes. The results demonstrate that the control molecule exhibited a relatively higher beta-factor profile, indicating potential flexibility as compared to the novel complexes, as seen in Figure 5B.

#### 2.5.3. SASA and Ligand RMSD

A lower ligand RMSD demonstrates better stability, while a higher value shows deviation in the ligand structure over the simulation time [20]. Among the lead complexes, lead-1 emerged as the most stable ligand, followed by the control molecule, as shown in Figure S2. The analysis of solvent-accessible surface area (SASA) demonstrates the exposure of the protein’s surface region to solvent. The plot illustration in Figure S2 indicates that the control molecule remained the most compact, as compared to the novel complexes, which were slightly exposed to the solvent.

### 2.6. Principal Component Analysis (PCA) and Free-Energy Landscape (FEL) Analysis

**Figure 6:**
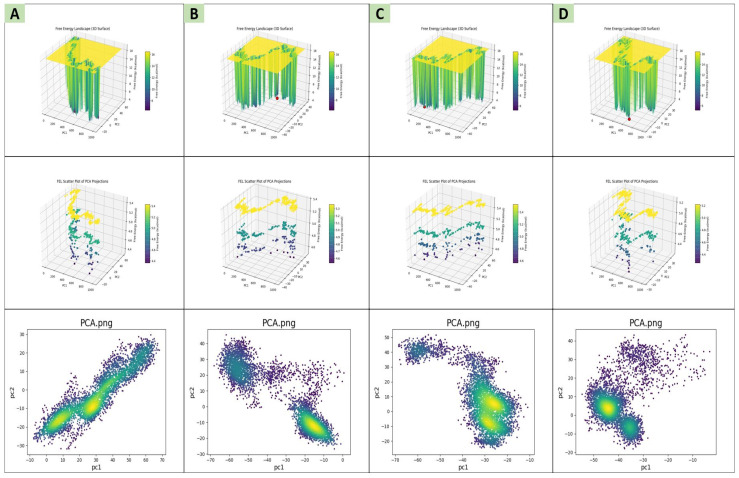
Comparative PCA and FEL plot evaluations of lead complexes, designated as lead-1 (**A**), lead-2 (**B**), lead-3 (**C**), and the control (**D**). The top row represents 3D-FEL, the middle row illustrates scattered PCA, and the bottom row shows two-dimensional PCA. The red dot shows energy minima for the complexes.

The function of a protein and the rigidity that is specifically needed on the binding side are determined significantly by its conformation. The covariance matrix was utilized to analyze large-scale motions during simulation [21]. Principal component analysis (PCA) was performed to determine the major conformations present during the simulation, and the overall flexibility was calculated using PC1 and PC2 parameters [22]. Figure 6 shows PC1 and PC2 projections for the three docked complexes, and the FEL was analyzed in order to provide information about their conformational stability [23]. With reduced free-energy minima, there is increased conformational stability [24]. Figure 6 also shows a map of the PCA from the three lead complexes compared to the control, color-coded from yellow, exhibiting a high-density area, to purple, low-density regions. Lead-1 (Figure 6A) demonstrated a distinct variation cluster and a good FEL, occupying the firm conformation. Additionally, lead-2 (Figure 6B) exhibited two distinct clusters with an uneven FEL (multiple regions), displaying the occurrence of conformational changes. Lead-3 (Figure 6C) and the control (Figure 6D) showed an intermediate profile, indicating moderate conformational shifts.

### 2.7. RDF Assessment

**Figure 7:**
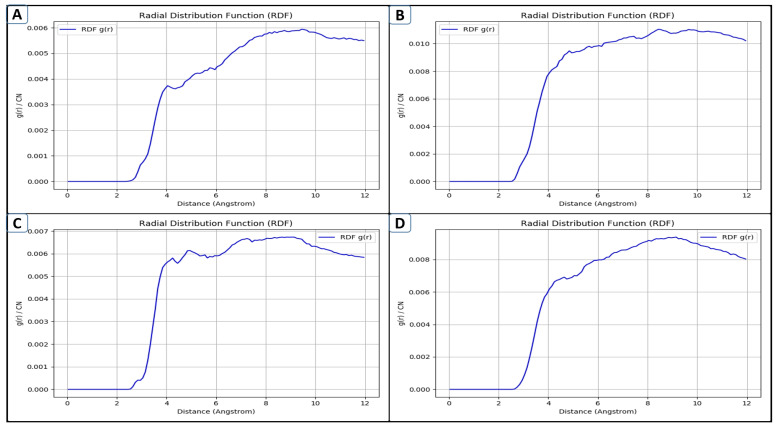
Radial distribution function plot of complexes, designated as lead-1 (**A**), lead-2 (**B**), lead-3 (**C**), and the control (**D**).

The typical radial packing of atoms in a system is expressed by the radial distribution function (RDF), which is a number that is calculated by regularizing graphs of atom-pair distances [25]. It helps determine the number of water molecules that are connected to a metal ion in the active site of a protein, for example, or with figuring out how well atoms coordinate. The solvent-aided binding process can be clarified using the RDF. It has also been extensively utilized for understanding how the atoms of tiny compounds interact with one another. A more dispersed atomic structure is linked to an increase in RDF frequency [26]. Comparing that computational method with a more thorough experimental investigation is made easier by this technique. Figure 7 depicts the RDF analyses of three lead complexes and the control molecule. Upon analyzing and assessing the plot, it is apparent that lead-2 (B) exhibited the most favorable binding and local structuring, with a tightly packed structure in the active pocket of GuaB. The control (D), on the other hand, ranked second, showing fairly stable findings with a peak around 5 Å and a smooth decline towards a plateau. Lead-1 (A) and lead-3 (C) were seen to have moderately ordered structures with broader and lower peaks, possibly suggesting a loosely packed/low-density structure inside the active region, as depicted in Figure 7.

### 2.8. Hydrogen Bonds Calculation

In computer-based drug discovery, the calculation of hydrogen bonds has an important influence on molecule recognition, binding affinity, and drug-like properties, including oral bioavailability and target affinity; therefore, understanding and controlling hydrogen-bonding interactions is vital for creating efficient drug delivery systems [27]. Table 4 offers detailed information about each compound’s h-bond interaction efficiency and binding potential. The table includes a number of parameters, including acceptor, donor and donorH, quantity of frames, frac, average distance, and average angle. The h-bonding characteristics of the lead compounds and the control are shown in the given table below. The total number of frames, average bond distance, average angle, and occupancy % were used to analyze the compounds’ interaction with GuaB. The H-bond calculation indicates that lead-2 emerged as the most stable complex, based on it having the highest number of frames (460) and contacts (13) among the other two lead GuaB complexes. The complex mentioned above formed hydrogen bonds with LIG_336@O1 and ASN_163@HD22, with an average bond angle of 151.27 Å, suggesting a promising geometry and diversity of hydrogen bonds. Furthermore, lead-3 and the control exhibited a moderately favorable profile, with the total number of frames created being 280 and 228. The following is the ranking of the complexes according to their interaction profiles and hydrogen-bonding stability: lead-2 > lead-3 > control > lead-1.

### 2.9. DCCM Analyses

**Figure 8:**
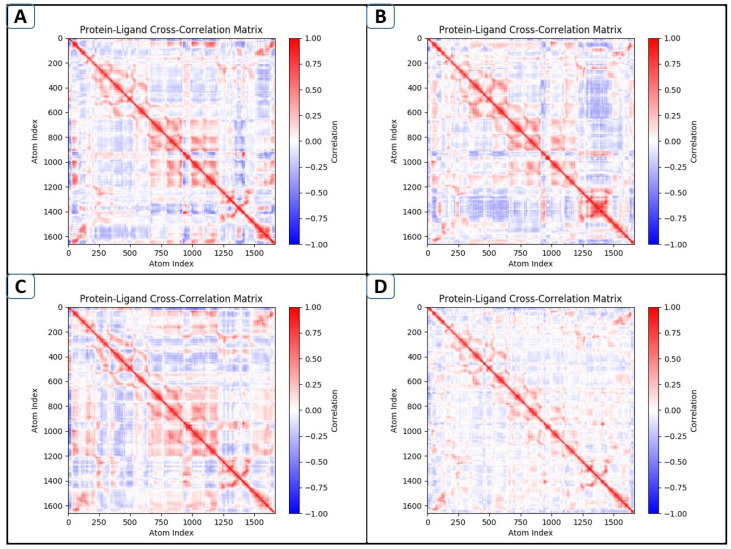
The dynamic cross-correlation matrices of GuaB complexes, designated as lead-1 (**A**), lead-2 (**B**), lead-3 (**C**), and the control (**D**).

The dynamic cross-correlation matrix (DCCM) analyses investigated the relative movements of structural domains to obtain a stable confirmation of the GuaB target following ligand interaction from MD trajectories [22]. The protein’s residue modifications were visible on the dynamic cross-correlation map. Figure 8 displays the positive as well as negative correlations of residue displacement throughout the simulation. Here, complexes of the GuaB (lead-1, lead-2, and lead-3) with the control (baseline) were calculated for correlated, anti-correlated, and non-correlated motion. The color scale shows the strength of the connection; red (+1) denotes a positive correlation while blue (−1) denotes a negative correlation. In the lead-1 complex (Figure 8A), we saw larger patches of strong positive and negative correlations scattered throughout the protein, indicative of more widespread cooperative and opposing motions. This implies a rather flexible binding environment. The highest-ranked compounds (Figure 8A–C) exhibited a significant decrease in large anti-correlated blue regions, especially in the areas of the residues around the ligand-binding cavity, as see in Figure 8. The reduction in long-range anti-correlated motions is reflective of a stabilization of the local structural arrangement on ligand binding. Figure 8B,C show enhanced red correlations in the ranges of the atomic indexes corresponding to ca. 400–700 and 1100–1300 and represent residues that form a substrate-recognition loop and adjacent alpha helices. These positively correlated motions indicate that binding of the ligand results in a promotion of synchronized structural fluctuations within the active site. The DCCM analysis for the control compound (Panel D) shows a relatively low overall intensity of correlation, suggesting a relatively reduced impact on global dynamics. Nevertheless, a small cluster of positive correlations remains proximal to the binding-site residues, indicating a moderate stabilization. In sum, the DCCM analysis shows that the three most potent compounds have more forceful localized correlated motions and suppress long-range anti-correlations more than the control. The pictorial analysis of all three leads and the control–GuaB complex demonstrates that lead-1 (Figure 8A) has high anti-correlated movements, suggesting less stable binding, whereas lead-2 was noted to have moderate correlation, suggesting intermediate binding with GuaB (Figure 8B). Lead-3 (Figure 8C) can be observed to have the most positive correlation (red color), signifying favorable stability. The control (Figure 8D), utilized as a baseline compound, showed weak and non-specific correlations, indicating Figure 8. decoupling, as displayed in Figure 8.

### 2.10. Salt-Bridge Analysis

**Figure 9:**
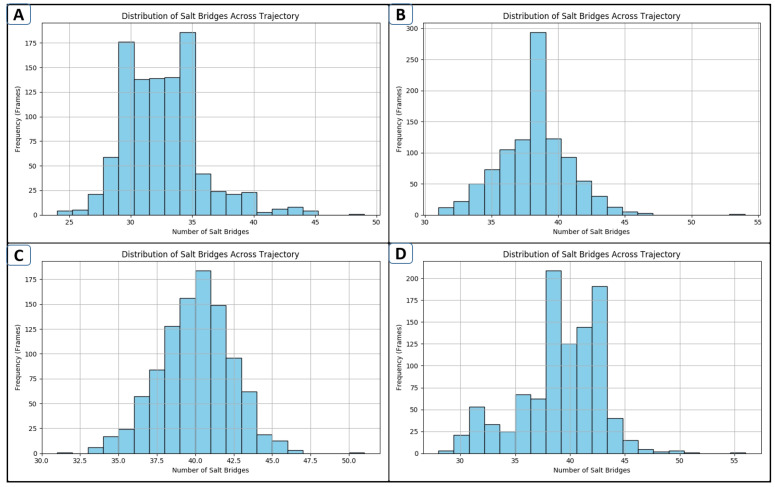
Salt-bridge distribution histogram of the complexes: lead-1 (**A**), lead-2 (**B**), lead-3 (**C**), and the control (**D**).

Salt bridges are stronger than simple hydrogen bonds because they are non-covalent interactions that combine electrostatic attraction between oppositely charged groups of molecules or atoms with hydrogen bonding [28]. In proteins, salt bridges are most commonly observed between negatively charged acidic residues (Asp or Glu) and positively charged basic residues (Lys or Arg). It has been shown that salt bridges contribute to protein–protein interactions, folding, recognition, conformational stability, and overall stability. The number of salt bridges directly correlates with protein stability. Sharper peaks indicate consistent performance, while bimodal peaks suggest flexibility, fluctuations, and transitions between states. The analysis of lead-1 (Figure 9A) shows bimodal peaks ranging from 25 to 45, indicating broader peaks that reflect dynamic shifts and flexibility. Conversely, lead-2 (Figure 9B) displays salt bridges between residues 32 and 45, with a peak centered at residue 39, suggesting that lead-2 is likely a stable structure. Additionally, lead-3 (Figure 9C) demonstrates electrostatic interactions between residues 32 and 50, with peaks near 40–44, indicating greater stability among the three leads. The control (Figure 9D) was characterized by low–moderate stability, with dynamical shifts and inconsistencies in salt-bridge formation (Figure 9). Salt bridges formed between aspartate/glutamate and lysine/arginine residues within the catalytic loop and the substrate-reception region had a persistence of more than 70% of the simulation frames in both lead-1 and lead-2 complexes. These electrostatic contacts are assumed to stabilize local secondary structural elements and many disturbances of conformation [29]. Ligands that strengthen these parametric electrostatic nodes can lead to an enhancement of stability in the functional architecture of the protein and, ultimately, an increase in binding affinity. The stronger persistence of distinct salt-bridge clusters in lead-1 and lead-2 is a support of their potential use as better stabilizers of the target protein.

### 2.11. Secondary-Structure Evaluation at the Binding of a Ligand

Secondary-structure modeling highlights the structural basis of GuaB functions [30]. Figure S3 shows plots of the secondary structures for the three protein–ligand complexes as well as for the control molecule. Analysis was directed towards conformational changes due to ligand binding. Within Figure S3, extended beta sheets are also shown in comparison to the percentage of helix (alpha) and other structures (loops, turns, and coils). Results suggest that compound C (lead-3) caused little disorder, indicating that it formed a better structure when bound.

### 2.12. MMGB/PBSA, Along with Entropy Energy Estimation

The binding free energies for several trajectories were calculated based on MD simulation using the MM/GBSA and MM/PBSA approaches [31]. Summaries are given in Table 5, in which more negative values represent greater compound stability. Compared to other complexes, lead-1 had lower binding energy scores of −134.96 kcal/mol (MM/GBSA) and −146.65 kcal/mol (MM/PBSA). The compounds, designated as lead-2 and lead-3, and the control exhibit MMGBSA scores of –125.74 kcal/mol, −112.62 kcal/mol, and −127.54 kcal/mol, respectively. On the contrary, the energy randomness as well as energy disorders of different states and processes are captured by the entropy energy. The viability of the reaction can be impacted by entropy energy changes and irregularities. The largest negative number, 12 kcal/mol for lead-1, among the values shown in Table 5 indicates the lowest entropy and, consequently, the most stability.

## 3. Discussion

The first pathogen on the top list of priority pathogens for novel antibiotics to be designated as a “red-alert” human pathogen is the *Acinetobacter* species, specifically *A. baumannii* [32]. The most common biofilm-associated diseases caused by the newly discovered global antibiotic-resistant Gram-negative bacterium *A. baumannii* include ventilator-linked pneumonia as well as catheter-associated infections, each of which shows resistance to antibiotics [33]. Due to its ability to produce mechanisms of antibiotic resistance, the bacteria may grow in hospital environments, which promotes the spread of multidrug-resistant strains around the world. Intensive care units (ICUs) have the greatest number of *Acinetobacter* infections, even though these diseases are rapidly spreading throughout hospital settings worldwide [7]. The urgent need for novel therapies to combat AMR is increasing globally; however, traditional drug development is expensive and time-consuming [6]. On the other hand, computer-aided drug design (CADD) is a computational approach that utilizes different algorithms to predict promising drug/compounds against a target protein. One of the biggest advantages of CADD is its cost-effectiveness and prompt nature. By utilizing this computer-based strategy, the compounds can be modified, analyzed, and optimized for enhanced function.

The multidrug-resistant (MDR) bacteria that cause *A. baumannii* infections are prevalent in healthcare environments and can cause life-threatening illnesses such as bloodstream infections, pneumonia, urinary tract infections, and wound infections [34]. The current study was designed to combat the aforementioned bacterial infections by targeting GuaB. Inosine 5′-monophosphate dehydrogenase (IMPDH), known as GuaB, is an enzyme linked to de novo guanine biosynthesis [8]. The study utilized different machine learning algorithms for the identification of active and inactive compounds, along with molecular docking and MD simulation. The ML models used were SVM, RF, KNN, and XGBoost. All models, however, performed well based on the results of the hold-out tests, with the SVM and KNN both receiving the highest performance score of 0.98. Meanwhile, the RF and XGBoost demonstrated dependable performance, with AUC ratings of 0.95 and 0.93, respectively, and reasonably high accuracy. It demonstrates that the RF model is the most dependable of the models that were examined because it provides a good equilibrium between forecasting true negative (inactive) and true positive (active) compounds. The library, consisting of 200 compounds, was virtually screened against the target enzyme, GuaB, via molecular docking analysis. The study ranked the promising compounds based on the lowest binding affinity score. The docking results were plotted and displayed in Figure S1. The three compounds selected were lead-1, lead-2, and lead-3, with the docking scores of −8.1, −7.6, and −7.5, measured in kcal/mol, respectively. The control was utilized as a baseline drug, showing the docking score of −7.5 kcal/mol. Following the development of reliable models [35], molecular docking was carried out on a library to determine favorable lead compounds. Molecular docking was employed to calculate the interaction conformations of these chosen lead compounds after the 3D structure of the Guab protein was arranged. The chosen compounds underwent MD simulation after being further examined for ADME characteristics. Furthermore, the three leads satisfied the Lipinski rule of five standard criteria and were deemed to be drug-like. A 200 ns MD simulation was carried out on three GuaB–compound complexes, and the mean RMSDs noted were as follows: lead-1 (2.55 Å), lead-2 (2.86 Å), and lead-3 (2.89 Å). The control was seen to have the mean RMSD score of 2.54 Å, slightly more stable than lead-1, followed by lead-2 and lead-3. Post-MD simulation analysis showed that lead-2 formed a dense h-bonds network, which initiated the highest number of frames (460). PCA and RDF analyses implied that lead-2 had obtained a stable energy state represented by a smooth FEL, while deep energy wells were observed in comparison with lead-1 and lead-2, which presented slightly weaker and rougher RDFs. Secondary-structure analyses revealed the formation of structural changes triggered by lead-2 in GuaB upon lead-2 binding that are indicative of increased stability. The salt-bridging histogram analysis showed that the greatest stability of electrostatic interactions was in lead-3, with values between 32 and 50, with peaks around the 40–44 residues, followed by lead-2 and lead-1. In contrast, the control exhibited relatively fair post-simulation findings. The MMPB/GBSA and entropy energy calculations identified lead-1 as the most robust compound with the least negative score, MMGBSA (134.96), MMPBSA (−123.07), and entropy energy (12), evaluated in kcal/mol.

One of the studies conducted by [35], aligned with our recent study, in which they utilized different computational approaches such as molecular docking and MD simulation against Mur enzymes, helps in peptidoglycan synthesis. The main disease is multidrug-resistant *Mycobacterium tuberculosis*. Based on the computer calculations, gallomyricitrin, the best-docked phytochemical molecule, inhibits the chosen target Mur enzymes by the formation of stable hydrogen bonds. The cross-correlation network, FEL, H-bond, vector movement, RMSD, Rg, RMSF, DCCM, and PCA, and the docked complexes of MurI, MurA, MurG, MurE, and MurC are found to be more stable during MD simulations than the docked complexes of MurB, MurD, MurF, and MurX. Gallomyricitrin also settled to the lowest global energy of Mur enzymes, according to FEL. By establishing hydrogen bonds, gallomyricitrin’s interactions within its binding regions were shown to be stable by the PCA, DCCM, vector movements, and binding free-energy data. There is a positive and negative connection between residues in various protein domains in Mur enzymes, according to the cross-correlation study.

One of the other studies, conducted on machine learning and molecular docking, was conducted by [36]. The study was based on potential natural epilepsy inhibitors discovered through the integration of virtual screening, molecular modeling, and ML techniques. Several machine learning algorithms, such as SVM, kNN, random forest, and naive Bayes (NB), were used in the research. These algorithms improved the ability to differentiate between inactive and active compounds by being used not solely for docking purposes, but also for key properties selection and execution. With an astounding 93.43 percent precision on both training as well as test datasets, the RF model outperformed the other machine learning algorithms that were investigated. After using this powerful RF model to sort through the 9000 phytochemicals in the collection, 180 possible inhibitors were identified. The active site of the S100B proteins was then docked with these active compounds. In the final analysis, the study stated that the use of machine learning and virtual screening in the study not only raises the possibility of discovering novel treatments for epilepsy but also highlights the revolutionary potential of these cutting-edge computational techniques for expediting and improving drug development procedures. Previous experimental studies have led to the understanding of important structural features and activity relationships of IMPDH inhibition [16,37,38], thus providing an empirical basis for the interpretation of our predictive analyses. Because biological systems are intricate and controlled by a number of significant factors, they have certain limitations even with the most advanced techniques, like CADD. A computer system cannot accurately mimic and model the complete biological system. A major problem that still affects the drug discovery method is target flexibility. Regarding the residues inside or close to the active site, the protein’s structure could be immobile or only slightly pliable. Additionally, because CADD relies on molecular models and algorithms that may not be entirely biologically sound, it may produce estimates that ignore important facets of protein–ligand interactions. The study’s findings suggest that extensive testing needs to be performed to assess the reliability and efficacy of the best compounds identified through in silico research. The in silico study has certain limitations, even though it has a lot of potential. Computational accuracy is mostly influenced by the simulation parameters and the protein’s structural quality. Docking studies may fail to account for solvent effects and protein flexibility, which could result in false positive results. To confirm and authorize the inhibitors’ abilities as drug development leads, an experimental investigation is required.

## 4. Materials and Methods

**Figure 10:**
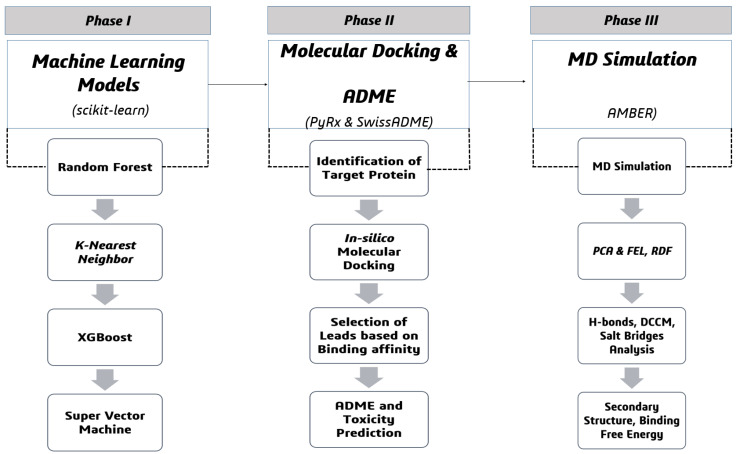
The overall workflow of the current computational study conducted in three phases: Phase I (machine learning models), Phase II (molecular docking and ADME), and Phase III (MD simulation).

The current research begins with the identification of active and inactive compounds from the CheMBL database in Phase I using a set of supervised machine learning algorithms, namely, random forests, k-nearest neighbors, XGBoost, and support vector machines for predictive classification. Phase II of the computational workflow is the identification of target proteins (GuaB), molecular docking studies to measure binding affinities, selection of candidate hits, and comprehensive ADME profiling for the evaluation of pharmacokinetic attributes. Finally, Phase III includes molecular dynamics (MD) simulations and subsequent trajectory-guided studies, including hydrogen-bond characterization, radial distribution functions (RDFs), dynamic cross-correlation matrices (DCCMs), principal component analysis (PCA), free-energy landscape (FEL) mapping, salt-bridge and secondary-structure assessments, as well as binding free energy calculations to evaluate the stability and dynamics of ligand–protein complexes, as seen in Figure 10.

### 4.1. Machine Learning-Guided Drug Design

#### 4.1.1. Dataset Preparation and Cleaning

A database of bioactive compounds inhibiting GuaB has been selected from the ChEMBL database. Using this resource, a total of 247 molecules associated with the GuaB therapeutic target in the context of antibiotic resistance were obtained. Of these, 153 molecules were given a binary label 1 for activity; decoy compounds were given the label 0 for inactivity [39]. The half-maximal inhibitory concentration (IC50) values were used as an indicator for measuring the biological activities of the compounds [40]. Duplicate entries that had identical SMILES representations were removed. If there were multiple activity measures for duplicate substances, then the endpoint for each molecule was the lowest IC50 value [41].

#### 4.1.2. Descriptors Calculations and Feature Extraction

The compiled dataset consisting of compounds has been loaded into a Pandas DataFrame for further processing. The dataset was divided into two major categories, namely, characteristics and the target variable. The target variable represented the activity state of each molecule that was encoded as “1” = active and “0” = inactive, while the characteristics were derived from the SMILES representations of the molecules. To enable model training and validation, the data were then split into training and test subsets using the train_test_split function of Scikit-learn, which yields the same number of active and inactive compounds in each subset [42]. Fingerprints and physicochemical descriptors were calculated using the RDKit Morgan algorithm.

#### 4.1.3. Conducting Objective Model Performance Testing

The congruence, validity, and generalization performance of a classification model need to be rigorously evaluated to guarantee a classification model’s substantive utility [43]. Nevertheless, well-established performance indicators are normally employed. Sensitivity (also known as TP), which measures the model’s ability to identify positive instances, is to be understood as a concept of probability. The processed data were then split into training and testing sets, the models were trained on the training data, and finally evaluated using a 5-fold cross-validation strategy in order to minimize the reliance on a given data configuration [44]. The hyperparameters of all models were optimized using Scikit-learn’s GridSearchCV function, which leaves no stone unturned by exhaustively searching an estimator’s parameter space [45]. Contrary to model parameters that are estimated in the training process, hyperparameters are parameters that are determined in advance and have a massive impact on how the models behave. The GridSearch process systematically explores every possible combination in a defined hyperparameter space and selects the best configuration, which in turn gives the best performance from the models. Model evaluation included the recall, accuracy, precision, F1-score, and Area under the receiver operating characteristic curve (AUC) [46]. A receiver operating characteristic (ROC) curve was also calculated, which visually shows the discriminative performance of the model for all values of decision thresholds [47]. The cumulative performance over the entire range of thresholds was captured by the area under the receiver-operating characteristic (AUC-ROC) statistic, which gives an overall indication of the model’s ability to discriminate between two groups, in particular, between active and inactive compounds [48].

#### 4.1.4. Machine Learning (ML) Model Development and Training

ML algorithms that divided compounds into active and inactive categories were trained using the processed dataset. The algorithms used for this comprised random forest (RF), XGBoost, k-nearest neighbors (KNNs), as well as support vector machine (SVM) [49]. Each model was built, adjusted, and validated using the Scikit-learn toolkit alongside cross-validation techniques [50].

##### K-Nearest Neighbors (KNNs)

KNN is a parameter-free algorithm that examines all the available data and classifies the new observations using a pre-specified proximity or a similarity function [51]. The present study is about the implementation of KNNs in the Scikit Learn platform. The algorithm was implemented using the KNeighborsClassifier () function, and the number of neighbors was optimized as it varied from one to ten using a grid search.

##### Support Vector Machine (SVM)

SVMs are a versatile and flexible model among machine learning models. The model can be used for regression analysis and also to perform linear as well as non-linear classification [52]. Through building of hyperplane in high-dimensional space, it performs a separation of the classes. The SVM was implemented with the gamma parameter set to “scale” using the method of Scikit-Learn’s SVC method. Low values of this parameter imply a weaker influence of distant training instances, while high values imply a more sensitive influence of nearby instances, thereby modulating the effect of individual training samples. In line with the existing literature, the “scale” setting was used to transform the gamma value in accordance with the dimensionality of the feature set [36]. Both linear and radial basis function (RBF) kernels were submitted to the grid search technique to optimize the corresponding hyperparameters [53].

##### Random Forest (RF)

RF is a powerful machine learning technique for use in classification and regression. In MBDT, a large number of DTs are employed in the training phase, and the class having the mode of individual predictions is chosen for the classification problems [54]. In regression problems, the total estimate is obtained by combining the predictions of all trees. The Scikit-Learn Random Forest classifier function was used to obtain the model.

##### XGBoost

One of the ML algorithms that fall under the ensemble learning family is XGBoost. This makes use of regularization and boosting to build a robust and accurate prediction model [55]. This training approach, consisting of successively fitting a series of weak learners (usually decision trees), where each learner is selected to correct the errors of all the preceding ones, is called “boosting” [56].

Compounds were selected for docking based on their ML-predicted activity probability. The study experimented with different levels (0.70, 0.75, and 0.80) and selected a primary cutoff of 0.80 to prioritize high-confidence candidates and to avoid losing borderline high-scoring molecules.

### 4.2. Molecular Docking Phase

#### 4.2.1. Identification and Preprocessing of the Target Receptor

The three-dimensional structure of the GuaB protein, serving as the therapeutic target for treating infections associated with *Acinetobacter baumannii*, was taken from the RCSB Protein Data Bank (PDB) (https://www.rcsb.org/?ref=nav_home, accessed on 25 October 2025) accessed on 8 June 2025 [57]. Among the several peptide chains making up the selected structure (PDB ID: 9C4M; resolution: 2.48 Å; organism: *A. baumannii*; determination method: X-ray diffraction), the receptor chain A was selected as the target for this study. Docking was first preceded by the removal of co-bound ligands as well as extraneous water molecules. Additionally, using the Discovery Studio Visualizer [58], polar hydrogen atoms were inserted.

#### 4.2.2. Library Preparation and Molecular Docking

To enable in-depth molecular interaction studies, the 153 compounds, which were determined to be active, were docked into the active site of the GuaB protein using a docking tool. These were carried out using the PyRx tool v0.8, an AutoDock Vina [59]. Afterwards, the target protein Guab was loaded into the respective docking software. The structural details of protein–ligand complexes, including the binding affinity of a specific ligand–protein combination, can be determined via molecular docking techniques [15]. AutoDock Vina was used for molecular docking analysis. A calculated mixture of various energy terms, such as van der Waals, electrostatics, hydrogen bonds, and restraint violation energies, provides the basis of the scoring function [60]. Hydrogen bonds and important residue interactions can be identified by visualizing the interactions between GuaB and the compounds using PyMol v2.4.5 (accessed on 15 June 2025) and Discovery Studio v2025 (accessed on 15 June 2025)[35].

#### 4.2.3. ADME Features Prediction

Drug discovery relies heavily on the prediction of ADME (absorption, distribution, metabolism, and excretion) features, since many favorable compounds cannot be developed into a drug during development because of adverse reactions or insufficient pharmacokinetics [61]. An in silico assessment was performed of the selected compounds’ drug-likeness to filter candidates with undesirable characteristics primarily in the development process. Several web-based tools, including SwissADME (https://www.swissadme.ch/) accessed on 20 June 2025, were employed to evaluate the pharmacokinetic characteristics [62].

#### 4.2.4. Molecular Dynamics (MD) Simulation

Molecular dynamics simulations were performed using AMBER v16 and 22, where the parametrization of proteins was performed using the ff19SB force field, and the representation of the ligands was performed using the GAFF2 force field [63]. Before system preparation, ligand parameters were generated using AM1-BCC charge models with the Antechamber module, thereby ensuring compatibility with the GAFF2 parameter set [64]. Each protein–ligand complex was placed inside an orthorhombic simulation box and explicitly solvated using the OPC water model with excellent dielectric and dynamical characteristics compared to the conventional TIP3P [65]. The solvation box was built such that it would have a minimum buffer distance of 10 Å around any molecule (solute) atom and the box boundary. Charge neutrality was achieved by the addition of appropriate numbers of Na+ or Cl- counterions, and subsequent adjustment of the ionic strength to have a physiological concentration when required [66]. A cutoff of 10 Å was used for short-distance non-bonded interactions [67]. Protein–ligand complexes were subjected to a multi-step energy minimization procedure consisting of (i) restrained minimization of solvent and ions, followed by (ii) unrestrained minimization of the entire system to reduce steric clashes and unfavorable contacts [68]. The minimized systems were then heated from 0 to 300 K in 100 ps under the NVT ensemble with a weak restraint on the position of the heavy atoms. Subsequent equilibration was performed under the NPT ensemble for 500 ps, to allow density stabilization [69]. Thereafter, 200 ns production MD simulations were performed for each complex in the NPT ensemble at 300 K and 1 atm. Temperature control was performed using Langevin dynamics at a collision frequency of 2 ps-1, while pressure control was performed using a Monte-Carlo barostat [70]. SHAKE constraints were added on all bonds involving hydrogen atoms, allowing the use of the 2 fs integration timestep [71]. The post-simulation analyses were performed and plotted using Python 3.7 [72].

### 4.3. Post-Simulation Analysis

#### 4.3.1. PCA and FEL

Principal component analysis (PCA), also known as essential dynamics (ED), is a dimensional reduction method used to explain dominant motional variations in proteins in the course of the simulations [35]. It was used to find important conformational changes and the dynamic behavior of the protein–ligand complex [11]. The MD trajectory data with PCA and FEL visualization of the molecules were processed using Python modules. A study of stability and conformational changes in the GuaB–ligand complex throughout the simulation trajectory was performed by FEL [73]. A relative energy minimum is a characteristic of these energy landscapes, which form the basis for quantifying the complex stability; in modeling these energy landscapes, one can distinguish important structural motifs, such as deep energy wells and transitional states [73].

#### 4.3.2. Radial Distribution Function (RDF)

The RDF is a measure of the atomic packaging at average distances by normalizing histograms of interatomic distances [74]. This metric can be used, for example, to calculate the number of water molecules that are coordinated to the metal ion or in the active site of a protein, or to calculate the degree of atomic coordination [75].

#### 4.3.3. An Analysis of H-Bonds

The formation of hydrogen bonds between the ligands and the residues in the GuaB active site during the simulation is important for estimating the strength and stability of binding [76]. Furthermore, MD simulations were used to analyze the hydrogen-bond pattern within the active site. Hydrogen bonding plays an important role in drug design to influence the structure, binding affinity, and solubility of the drug by regulating its interaction with the target protein [77].

#### 4.3.4. DCCM Analysis from MD Trajectories

The cross-correlation matrix is a three-dimensional observation of the pattern of the protein residue’s temporal correlations [22]. To explore the correlated motions within the docked complex residues, a DCCM investigation was carried out on the backbone Ca atom trajectory [78].

#### 4.3.5. Secondary-Structure Analysis

The secondary structure of the Guab was analyzed to find conformational changes induced upon binding of the ligand [79]. Such alterations can be seen in the alpha helix, beta sheet, coil, turns, or loops in the protein [30]. The analysis used trajectory data and Python for visualization of the secondary structure plot.

#### 4.3.6. Salt-Bridge Assessment

Salt bridges were computed using the trajectory data. Such analyses are indispensable in computational drug design because salt bridges play a special role in stabilizing protein structures, having consequences for molecular interactions which occur between oppositely charged amino acid residues [28]. Statistical analysis of salt bridges increases the understanding of the dynamics of the protein–ligand complex and its equilibrium by identifying key interactions that are required for drug binding [80]. A histogram of salt bridges was created using a Python module.

#### 4.3.7. Protein Solvent Environment: MMGB/PBSA and Entropy Energy Estimation

The molecular mechanics Poisson–Boltzmann surface area (MM/PBSA) and molecular mechanics generalized Born surface area (MM/GBSA) approaches can be used in combination with MD to calculate the binding-free-energy of protein–ligand complexes [81]. Calculations were performed with the extracted MD trajectories. Binding-free-energy provides a concise summary measurement of the biomolecular interactions; potential energy, polar-solvation energies, and non-polar-solvation energies are combined to give the total binding energy [31]. In addition, entropy energy estimation was used for the evaluation and disorder analysis of the protein–ligand system [82].

## 5. Conclusions

To identify potential GuaB receptor antagonists from the compound library, this study employed a multi-phase virtual screening approach utilizing machine learning. Based on key evaluation metrics such as Matthews’ correlation coefficient (MCC), ROC curve study, and accuracy (Q), all models created with different algorithms demonstrated strong predictive performance. After this screening, hits were subjected to molecular docking within the GuaB active site. Each lead binding conformation was used to compare docking results to the reference/control docked complex (−7.5 kcal/mol). Three top-scoring compounds were selected based on their docking scores and critical residue interactions within the GuaB active pocket. Molecular dynamics (MD) simulations, conducted over 200 ns, further validated these findings and assessed the stability of ligand–protein interactions relative to the control complex. Throughout the simulations, the novel leads exhibited notably stable binding behavior, with a consistent RMSD graph and reliable interactions. Additionally, post-simulation analyses—including RDF, hydrogen bonds, PCA, FEL, DCCM, salt bridges, and secondary-structure interactions—confirmed that the newly identified compounds are potent against GuaB. Their substantial binding affinities were further supported by free-energy estimations using MM-PBSA and MM-GBSA, indicating that these compounds could effectively inhibit GuaB activity. Consequently, based on current results, lead-2 and lead-3, followed by lead-1, appear to be promising candidates for developing GuaB-targeted antimicrobial resistance (AMR) therapies. Moreover, this validated machine learning model provides a valuable tool for discovering additional potential GuaB inhibitors with therapeutic relevance; however, further experimental investigation needs to be performed to explore their potential efficacy.

## Figures and Tables

**Table 1:** Information about the models’ performances (random forest, support vector machine, k-nearest neighbor, and XGBoost) on the test set. The different parameters have the following meanings. SE: sensitivity (true positive rate); SP: specificity (false positive rate); Q+: positive predictive value (precision); Q−: negative predictive value; ACC: accuracy; and MCC: Matthews’ correlation coefficient.

Dataset	Model	SE	SP	Q+	Q−	ACC	F1 Score	MCC
Testing Set	Random Forest	97	87	96	97	96	98	91
	Support Vector Machine	96	75	92	89	93	96	83
	K-Nearest Neighbor	95	75	92	95	93	96	83
	XGBoost	91	87	96	92	96	97	91
Validation Set	Random Forest	96	90	95	96	97	98	93
	Support Vector Machine	96	97	95	90	97	98	93
	K-Nearest Neighbor	96	83	95	90	96	98	93
	XGBoost	95	90	96	90	95	96	86

**Table 2:** Molecular docking findings of promising hit ChMBL compounds against GuaB–*A. baumannii*.

S. #	Compound Rank	Binding Affinity	Chemical Name	Chemical Structure
1.	Lead-1	−8.1 kcal/mol	3-(5-(3,4-dimethylphenyl)-1,2,4-triazolidin-3-yl)-4-hydroxybenzenesulfonamide	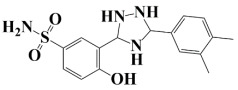
2.	Lead-2	−7.6 kcal/mol	3-carboxy-1-(2,4-difluorophenyl)-6-fluoro-5-methyl-7-(piperazin-1-yl)quinolin-1-ium-4-olate	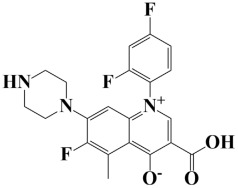
3.	Lead-3	−7.5 kcal/mol	2-(5-(3,4-dimethylphenyl)-1,2,4-triazolidin-3-yl)-4,6-dimethylphenol	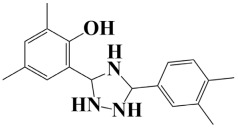
4.	Lead-4	−7.4 kcal/mol	3-carboxy-1-(2,4-difluorophenyl)-6-fluoro-7-(pyridin-1-ium-4-yl)-1,8-naphthyridin-1,8-diium-4-olate	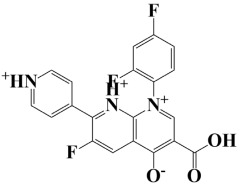
5.	Lead-5	−7.2 kcal/mol	6-(1-hydroxyethyl)-3-((5-((3-methyl-2,3-dihydro-1H-imidazol-1-yl)methyl)benzo[d]thiazol-3-ium-2-yl)thio)-7-oxo-1-azabicyclo[3.2.0]hepta-2,4-diene-2-carboxylate	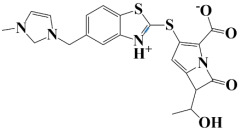
6.	Lead-6	−7.2 kcal/mol	2,4-dimethyl-6-(5-(4-methyl-3-(trifluoromethyl)phenyl)-1,2,4-triazolidin-3-yl)phenol	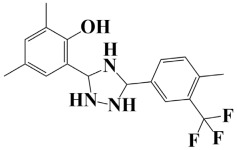
7.	Lead-7	−7.2 kcal/mol	3-carboxy-1-cyclopropyl-6-fluoro-7-(pyrazin-1,4-diium-1-yl)-1,8-naphthyridin-1,8-diium-4-olate	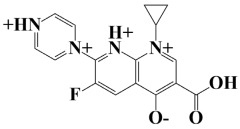
8.	Lead-8	−7.1 kcal/mol	((2-((2-carboxy-6-(1-hydroxyethyl)-7-oxo-1-azabicyclo[3.2.0]hepta-2,4-dien-3-yl)thio)ethyl)amino)methaniminium	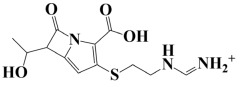
9.	Lead-9	−7 kcal/mol	7-(3-amino-1H-pyrrol-1-yl)-3-carboxy-1-(2,4-difluorophenyl)-6-fluoro-1,8-naphthyridin-1,8-diium-4-olate	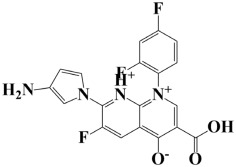
10.	Lead-10	−7 kcal/mol	7-(3-amino-1H-pyrrol-1-yl)-3-carboxy-1-(2,4-difluorophenyl)-6-fluoro-5-methylquinolin-1-ium-4-olate	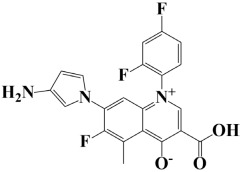
11.	Control	−7.5 kcal/mol	3-(hydroxymethyl)-6-((1-((4-methyl-3-morpholinophenyl)amino)-1-oxopropan-2-yl)amino)quinolin-1-ium	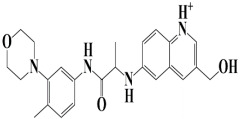

**Table 3:** The ADMET parameters of novel identified compounds, along with the control molecule.

ADME Properties							Toxicity Properties				
Compounds	G-I	Bioavailability Score		TPSA		Consensus Log Po/w	AMES Toxicity		hERG I Inhibitor		hERG II Inhibitor
Lead-1	High	0.55		124.86 Å^2^		0.93	No		No		No
Lead-2	High	0.55		79.51 Å^2^		2.13	No		No		No
Lead-2	High	0.55		56.32 Å^2^		3.08	No		No		No
Control	High	0.55		87.97 Å^2^		2.52	No		No		Yes
**Lipinski’s Rule of 5 Profile**											
**MW**			**MlogP**		**H-BA**			**H-BD**		**Lipinski**	
348.42 g/mol			1.21		7			5		Yes, 0 Violations	
417.38 g/mol			−1.28		7			2		Yes, 0 Violations	
297.39 g/mol			3.08		4			4		Yes, 0 Violations	
421.51 g/mol			3.46		3			4		Yes, 0 Violations	

Abbreviations: MW stands for molecular weight, HBA stands for hydrogen-bond acceptor, HBD stands for hydrogen-bond donor, GI stands for gastrointestinal absorption, and TPSA stands for topological polar surface area.

**Table 4:** The hydrogen bonds of lead GuaB complexes, along with the control.

Lead-1						
#Acceptor	DonorH	Donor	Frames	Frac	AvgDist	AvgAng
LIG_336@N3	GLY_200@H	GLY_200@N	129	0.129	2.9266	162.2301
HIE_137@ND1	LIG_336@H1	LIG_336@N2	14	0.014	2.8751	148.5278
THR_166@OG1	LIG_336@H2	LIG_336@O	8	0.008	2.8443	154.6046
HIE_137@ND1	LIG_336@H2	LIG_336@O	1	0.001	2.9215	161.8313
ALA_136@O	LIG_336@H1	LIG_336@N2	1	0.001	2.9384	155.4181
PRO_187@O	LIG_336@H1	LIG_336@N2	228	0.228	2.8765	157.7332
GLY_186@O	LIG_336@HN	LIG_336@N3	63	0.063	2.8572	151.7532
ASP_12@OD2	LIG_336@H	LIG_336@N	9	0.009	2.7906	149.4962
MET_201@O	LIG_336@HN	LIG_336@N3	1	0.001	2.8301	166.6044
ARG_194@NH1	LIG_336@H2	LIG_336@O2	1	0.001	2.9643	142.3263

**Table 5:** MMGB/PBSA and entropy energy estimation of the three lead compounds, with the control as a baseline drug.

Technique	Energy Section	Lead-1	Entropy	Lead-2	Entropy	Lead-3	Entropy	Control	Entropy
MMGBSA	Van der Waals Energy (kcal/mol)	−111.64 (±7.21)	12	−98.71 (±5.36)	11.4	−96.37 (±5.01)	11.5	−100.88 (±6.07)	11.8
	Electrostatic Energy (kcal/mol)	−35.01 (±5.37)		−38.49 (±4.36)		−32.52 (±3.67)		−38.19 (±6.50)	
	Polar-Solvation Energy (SE) (kcal/mol)	21.36 (±3.08)		19.47 (±1.89)		24.34 (±2.34)		22.09 (±2.05)	
	Non-Polar SE (kcal/mol)	−9.67 (±1.63)		−8.01 (±0.56)		−8.07 (±0.55)		−10.56 (±0.61)	
	Gas Phase Energy (kcal/mol)	−146.65 (±8.44)		−137.2 (±8.69)		−128.89 (±7.80)		−139.07 (±9.03)	
	Total Binding Energy (kcal/mol)	−134.96 (±7.56)		−125.74 (±7.00)		−112.62 (±6.30)		−127.54 (±6.21)	
MMPBSA	Van der Waals Energy (kcal/mol)	−111.64 (±7.21)		−98.71 (±5.36)		−96.37 (±5.01)		−100.88 (±6.07)	
	Electrostatic Energy (kcal/mol)	−35.01 (±5.37)		−38.49 (±4.36)		−32.52 (±3.67)		−38.19 (±6.50)	
	Polar Salvation Energy (SE) (kcal/mol)	30.66 (±3.01)		29.78 (±3.19)		26.70 (±3.50)		28.90 (±3.45)	
	Non-Polar SE (kcal/mol)	−7.08 (±0.69)		−8.11 (±0.48)		−7.58 (±0.59)		−9.73 (±1.28)	
	Gas Phase Energy (kcal/mol)	−146.65 (±8.44)		−137.2 (±8.69)		−128.89 (±8.09)		−139.07 (±9.79)	
	**Total** (kcal/mol)	−123.07 (±7.13)		−115.53 (±7.59)		−109.77 (±6.58)		−119.9 (±6.37)	

## Data Availability

The data generated in the work are presented either in the manuscript or its Supplementary File.

## Supplementary Materials

The following supporting information can be downloaded at: https://www.mdpi.com/article/10.3390/ph18121842/s1, Figure S1: Heatmap of molecular docking plot (Histogram and Scattered plot), Figure S2: Ligand RMSD (A) and SASA (B) of three leads and control, Figure S3: The secondary structure analyses of three lead complexes, as lead-1 (A), lead-2 (B), lead-3 (C), and control (D).
