# Supplementary material for: Decoding GuaB: Machine Learning-Powered Discovery of Enzyme Inhibitors Against the Superbug Acinetobacter baumannii
Source: Pharmaceuticals (Basel). 2025 Dec 2;18(12):1842. doi: 10.3390/ph18121842 (PMC12735981; doi:10.3390/ph18121842)
Supplement: Supplementary file 1 [file pharmaceuticals-18-01842-s001.zip › pharmaceuticals-3979396-supplementary.pdf]

Supplementary File

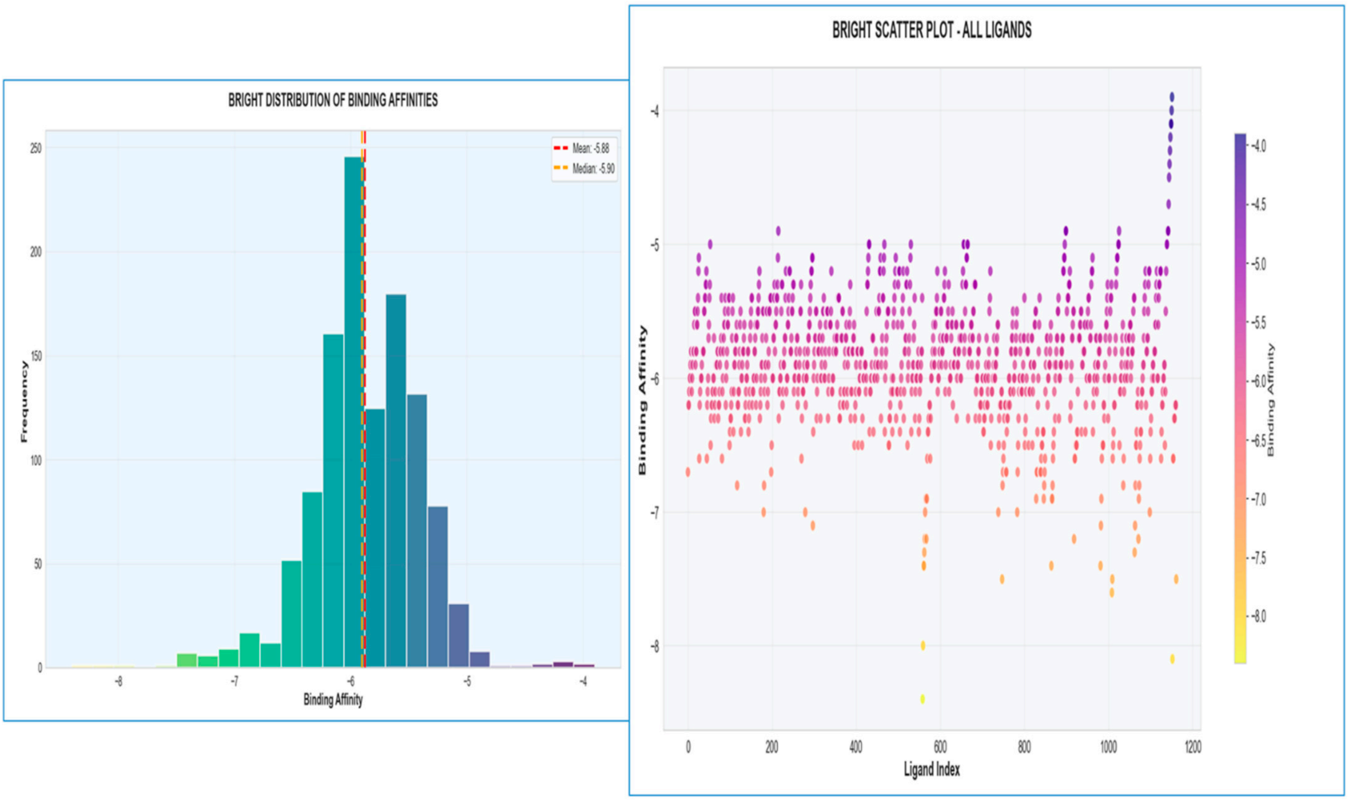

Figure S1: Heatmap of molecular docking plot (Histogram and Scattered plot)

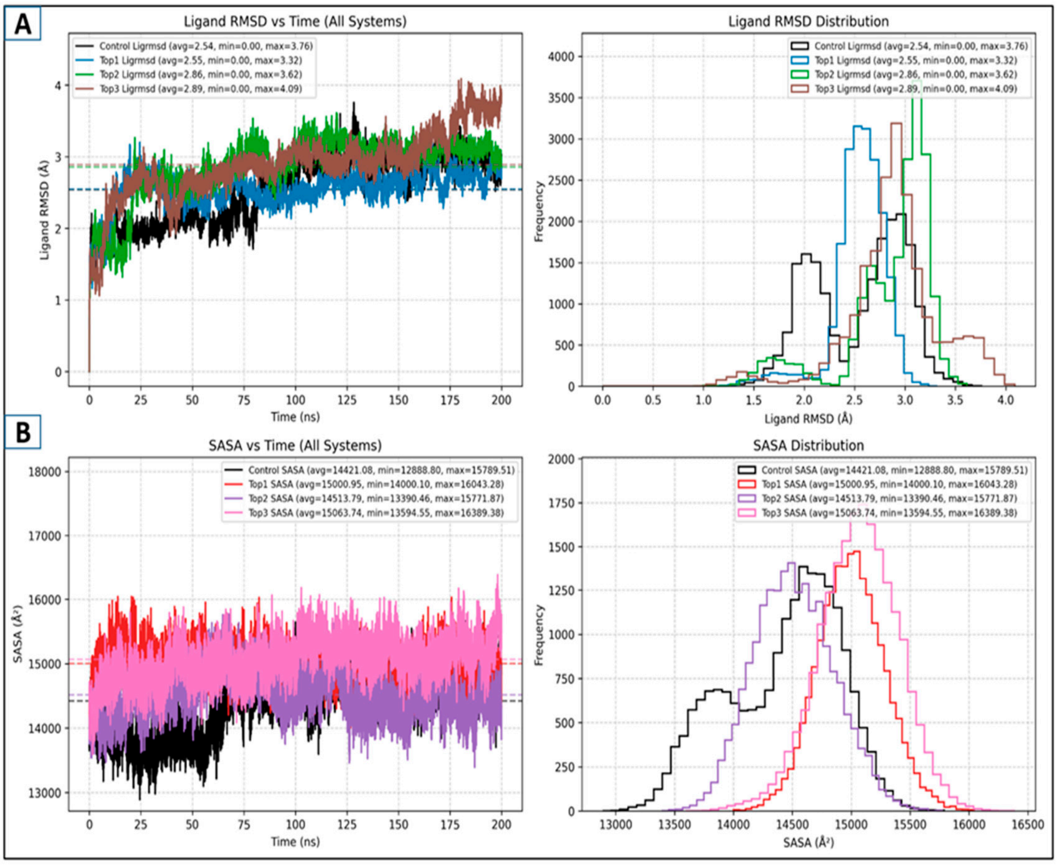

Figure S2: Ligand RMSD (A) and SASA (B) of three leads and control.

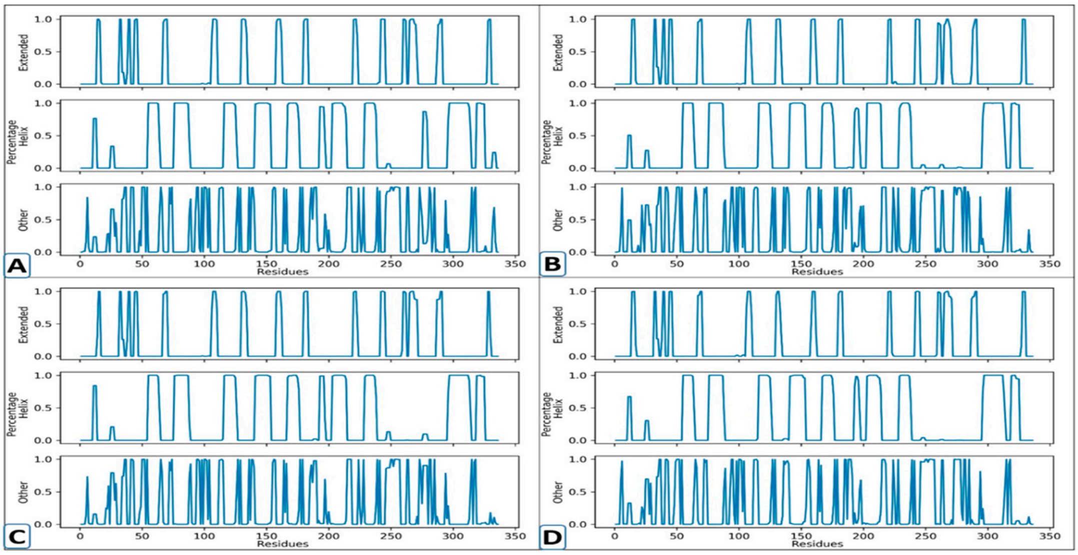

Figure S3: The secondary structure analyses of three lead complexes, as lead-1 (A), lead-2 (B), lead-3 (C), and control (D).
